# Muscle Oxygenation Response During Duplicate Sprints in Professional Football Players: An Original Investigation

**DOI:** 10.3390/muscles4040054

**Published:** 2025-11-11

**Authors:** Andrew Usher, John Babraj, Adam Younger

**Affiliations:** Department of Sport and Exercise Science, Abertay University, Bell St., Dundee DD1 1HG, UK; j.babraj@abertay.ac.uk (J.B.);

**Keywords:** NIRS, soccer, muscle oxygenation

## Abstract

Football requires repeated sprint ability for game-changing moments; however, the demand on the skeletal muscles is unknown. The aim of the current study was to determine the muscle oxygen response during duplicate sprints in professional footballers. Eight male professional footballers (age: 29 ± 5 y; height: 181 ± 8 cm; weight: 78 ± 8 kg) were recruited. Participants wore their normal GPS unit and completed their normal match warm-up before near-infrared monitors were attached to the rectus femoris and bicep femoris muscles. Participants then completed two 30 m sprints with 10 s of recovery, while GPS data and muscle oxygenation were recorded. Max speed was unaltered across the two sprints (s1: 8.4 ± 0.3 m.s^−1^; s2: 8.4 ± 0.4 m.s^−1^), but max acceleration (s1: 5.0 ± 1.5 m.s^−2^; s2: 3.7 ± 1.2 m.s^−2^) and time to max acceleration (s1: 1.0 ± 0.3 s; s2: 1.8 ± 0.8 s) were significantly different in sprint 2 compared with sprint 1. Change in muscle oxygenation was greater in the bicep femoris muscle than in the rectus femoris muscle in sprint 1 (right BF: 37.0 ± 14.7%; right RF: 23.4 ± 14.8%). Time to fast delay was longer in sprint 2 than in sprint 1 in the bicep femoris muscle (right BFs1: 1.6 ± 1.2 s; right BFs2: 5.2 ± 2.3 s), reflecting different recovery kinetics in the two muscles. During duplicate sprints there is a difference in oxygen response between the two muscles, and the overall recovery of the bicep femoris is much slower. This suggests poorer conditioning of the bicep femoris muscle, which may impact injury risk in professional football players.

## 1. Introduction

Football is an intermittent high-intensity sport consisting of repeated sprint activity across 90 min [1]. These high-speed linear running movements during a game have been strongly associated with positive game outcomes such as scoring a goal or shots on goal [2]. Typically, a sprint activity of 4–9 s duration occurs every 30–120 s during match play [2]. It has been highlighted that, over the last 15 years, the intensity of football match play has increased, with an ~1–3% increase in total sprint distance and an ~7–11% increase in total high-speed running distance [1]. The increase in game intensity has been associated with an annual increase in hamstring injury in professional football across the same period, with 70% of hamstring injuries occurring during linear high-speed running [3]. This suggests that we need a better understanding of the demands of linear high-speed running in professional football players.

Repeated sprint ability in football has traditionally been looked at based on speed decline across the sprint set [4]. Typically, in professional football players, 30 m sprint time is approx. 4 s with a decline of 3–7% in speed across 12 repetitions with 30 s recovery [4]. Repeated sprint ability has been shown to be associated with changes in blood lactate, with greater blood lactate accumulation post-test leading to a greater speed decrement [4]. Likewise, higher aerobic capacity assessed by the VO_2_ max score has been associated with lower average and total repeated sprint times [5]. Professional players have been shown to have lower average sprint times across 6 × 40 m sprints than amateur players with a similar VO_2_ max score due to faster oxygen on-kinetics, suggesting that the ability to quickly utilise oxygen is a key determinant of repeated sprint ability [6]. However, all these are measures of central performance and do not explore the demands placed on the muscle during repeated linear sprints.

Near-infrared spectroscopy offers the opportunity to explore muscle oxygenation during high-speed movements [7]. In team sports, vastus lateralis deoxygenation during repeated 4 s treadmill sprints has been shown to be strongly associated with sprint time [8]. In Spanish third-division football players, gastrocnemius muscle deoxygenation has been shown to be impeded after the fourth 20 m sprint when 20 s of recovery is given between each sprint [9]. In semi-professional football players, the extent of vastus lateralis SmO_2_ change is lower in the second sprint than in the first when 20 s recovery is given [10]. However, given the importance of the rectus femoris and bicep femoris to sprinting mechanics [11], it is crucial to gain a better understanding of the demand placed on these muscles during repeated sprint activities in professional footballers.

Football is a game that is determined by sprint performance, and the rectus femoris and bicep femoris muscles are the key muscles involved in sprinting. Therefore understanding the response of these muscles under continued sprint load is crucial to improve player and team performance. This study aimed to determine the impact of duplicate sprint activity on muscle deoxygenation of the rectus femoris and bicep femoris muscle. It was hypothesised that muscle deoxygenation change will be impeded in both muscles from sprint 1 to sprint 2.

## 2. Methods

Participants: 8 male professional football players (age: 29 ± 5 y; height: 181 ± 8 cm; weight: 78 ± 8 kg) consisting of 2 central defenders, 4 midfielders and 2 strikers were recruited for this study. The club had 5 central defenders, 10 midfielders and 5 strikers available for testing; therefore, 40% of the first team squad were involved in the study. All players were first team squad players with a minimum of 2 years’ experience as part of the first team squad. Any players with musculoskeletal stiffness or injury were excluded from the study.

Testing: GPS units were switched on prior to the session (Catapult vector core, Catapult Innovations, Melbourne, Australia), and participants wore the GPS units throughout, with data recorded at 10 Hz. The Catapult system has been shown to have good reliability for maximal sprint speed compared with timing gates [12]. Prior to testing, a 30 m linear course was measured (trundle wheel, Silverline tools, Somerset, UK) with a 5 m deceleration zone at either end. Participants completed their normal team-based soccer warm-up and then had NIRS devices (Moxy monitor, Fortiori Design LLC., Hutchison, MN, USA) taped to their rectus femoris and bicep femoris muscles. Muscle site was determined by palpation, and black tape was placed over the units to shield for light pollution. NIRS devices recorded data at 2 Hz, and data was captured through VO_2_ master app version 0.99 (VO_2_ Master Health Sensors Inc., Vernon, BC, Canada). There was a 275 ± 35 s gap between the end of warm-up and the 30 m sprint start. Participants were then instructed to sprint as fast as possible across the 30 m course and given a 10 s recovery before completing a second 30 m straight-line sprint. The average recovery time from peak speed in sprint one to the start of the second sprint was 12.7 ± 3.7 s.

**Figure 1:**
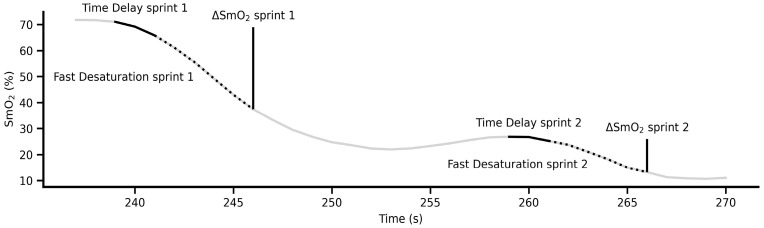
Muscle desaturation profile (black line represents initial time delay; dashed line represents fast desaturation; vertical line represents the change in SmO_2_ across the fast desaturation).

Data analysis: Raw data for GPS and NIRS was processed in Python software (Jupyter Lab version 4.3.4). NIRS data during the duplicate sprints was processed as described before [13] with time delay to fast desaturation, rate of fast desaturation and change in SmO_2_ across the sprint determined (Figure 1).

**Figure 2:**
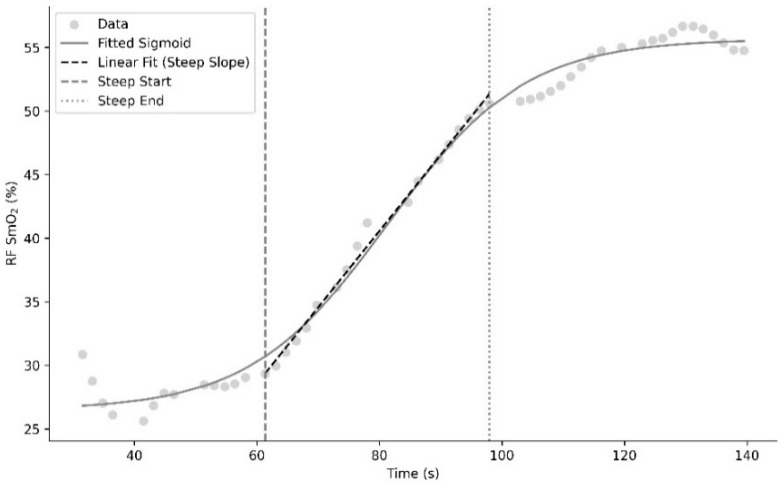
Example trace of analysis components for skeletal muscle SmO_2_ recovery.

Skeletal muscle recovery oxygenation after sprint 2 was modelled using a sigmoidal curve fit and linear regression as shown in Figure 2. A sigmoidal function was chosen, as the oxyhaemoglobin dissociation curve follows a sigmoidal pattern [14]. Briefly, the following sigmoidal function was fitted [15]:

L/(1 + np.exp(−k ∗ (x − x0))) + b

where L is the change in SmO_2_ across the curve; k is the rate constant describing steepness of the curve; x0 is the time to midpoint and b is the baseline SmO_2_ value (Figure 2). Goodness of fit for the recovery sigmoidal curve was right rectus femoris: 0.994 ± 0.005, left rectus femoris: 0.992 ± 0.006, right bicep femoris: 0.994 ± 0.004, left bicep femoris: 0.984 ± 0.014. A linear function was fitted across the steep region of recovery, with slope representing the rate of fast resaturation, and time to linear fast representing the delay from end of the second sprint to the start of the steep region (Figure 2).

Raw latitude, longitude and speed were taken from the GPS units. Max speed was determined as the maximum speed value in an individual sprint; distance was calculated using the Haversine formula:

d = 2r ∗ arcsin √(sin^2^(2Δlat) + cos(lat point 1) ∗ cos(lat point 2) ∗ sin^2^(2Δlon))

where Δlat = lat point 2 − lat point 1; Δlon = lon point 2 − lon point 1; r is Earth’s radius (6371 km).

Acceleration and deceleration were calculated as follows:

acc/dec = (speed point 2^2^ − speed point 1^2^)/(2 ∗ distance between points)

for average acceleration, point 1 was the starting point of the sprint and point 2 was at maximum speed of the sprint, and for max acceleration, the software iterated the calculation from starting point + 4 datapoints until max speed was reached and recorded the highest acceleration value within the sprint; for average deceleration, point 1 was maximum speed of the sprint and point 2 the minimum speed of the sprint occurring after the maximum value, and for max deceleration; the software iterated the calculation from starting point + 4 datapoints until max speed was reached and recorded the highest deceleration value within the sprint [16]. Metabolic power was calculated as described by Brochhagen J and Hoppe [17].

Statistical analysis: All data are reported as means ± standard deviation. All data was checked for skewness and kurtosis and was normally distributed. Running metrics were analysed using a paired-samples *t*-test to determine the difference between sprint 1 and sprint 2 with significance accepted at *p* ≤ 0.05. For running metrics, Cohen’s d effect was calculated with <0.25 as a trivial effect, 0.25–0.5 as a small effect, 0.5–1.0 as a moderate effect and >1.0 as a large effect (Rhea MR 2004). For sprint SmO_2_ changes, a 2 × 2 × 2 repeated measures ANOVA was used. If there was a significant effect or interaction effect (*p* ≤ 0.05), then Fisher’s LSD post hoc analysis was run to determine where differences occurred. For recovery, a 2 × 2 repeated measures ANOVA was used. If there was a significant effect or interaction effect (*p* ≤ 0.05), then Fisher’s LSD post hoc analysis was run to determine where differences occurred. Partial eta squared was recorded for each ANOVA, and effect size determined 0.02–0.13 as a small effect, 0.13–0.26 as a moderate effect and >0.26 as a large effect [18]. Multivariate linear regression and Kendall’s tau were used to explore linear and non-linear correlation between muscle oxygenation components and sprint performance.

## 3. Results

There was no significant difference in time for each 30 m sprint or in max speed during each sprint (*p* > 0.05; Table 1), with a trivial effect for both (d < 0.25). There was no significant difference in maximum or average deceleration between sprints (*p* > 0.05; Table 1); however, there was a small effect on maximum deceleration, which was greater in sprint 1 (d = 0.30), and a moderate effect for average deceleration, which was lower in sprint 1 (d = −0.76). There were significant differences between sprint 1 and 2 for maximum acceleration (*p* = 0.048; Table 1), time to maximum acceleration (*p* = 0.020; Table 1) and average acceleration (*p* = 0.048; Table 1), with a moderate effect for maximum acceleration (d = −0.84) and average acceleration (d = −0.84) and a large effect for time to maximum acceleration (d = 1.06).

There was no main effect for leg or muscle (*p* > 0.05; Table 2) for time delay, but there was a large effect for each (η^2^_p_ = 0.295, 0.127, respectively). There was a significant main effect for sprint (*p* = 0.050; Table 2) with a large effect (η^2^_p_ = 0.444) and a significant interaction effect for sprint × muscle (*p* = 0.014; Table 2) with a large effect (η^2^_p_ = 0.661). There was a significant difference in time delay for the right rectus femoris muscle compared with the left rectus femoris muscle in sprint 1 (*p* = 0.042; Table 2). There was a significant difference in time delay in sprint 2 compared with sprint 1 for the right bicep femoris muscle (*p* = 0.018; Table 2) and the left bicep femoris muscle (*p* = 0.012; Table 2).

There was no main effect for sprint, leg or muscle (*p* > 0.05; Table 2) for fast desaturation, but there was a large effect for each (η^2^_p_ = 0.168, 0.251, 0.115, respectively).

There was no main effect for sprint or leg (*p* > 0.05; Table 2) for change in SmO_2_, but there was a large effect for each (η^2^_p_ = 0.224, 0.219, respectively). There was a significant main effect for muscle (*p* = 0.042; Table 2) with a large effect (η^2^_p_ = 0.469). There was a significant difference in the change in SmO_2_ for the left rectus femoris muscle compared with the left bicep femoris muscle for sprint 1 (*p* = 0.027; Table 2) and for the right rectus femoris muscle compared with the right bicep femoris muscle for sprint 1 (*p* = 0.032; Table 2).

There was no main effect for leg (*p* > 0.05; Table 3) for k value, and there was a trivial effect (η^2^_p_ = 0.023). There was a significant main effect for muscle (*p* = 0.002; Table 3) with a large effect (η^2^_p_ = 0.783). There was a significant difference between the left rectus femoris muscle and the left bicep femoris muscle (*p* = 0.022; Table 3) and the right rectus femoris muscle and the right bicep femoris muscle (*p* = 0.023; Table 3).

There was no significant main effect for leg or muscle (*p* > 0.05; Table 3) for L value, although there was a moderate-to-large effect (η^2^_p_ = 0.219; 0.101, respectively).

There was no significant main effect for leg or muscle (*p* > 0.05; Table 3) for time to 50% recovery, although there was a moderate-to-large effect (η^2^_p_ = 0.285; 0.113, respectively).

There was no main effect for leg (*p* > 0.05; Table 3) for linear fast recovery rate, and there was a large effect (η^2^_p_ = 0.310). There was a significant main effect for muscle (*p* = 0.038; Table 3) with a large effect (η^2^_p_ = 0.495). Post hoc analysis could not detect significant difference.

There was no main effect for leg (*p* > 0.05; Table 3) for duration of linear response, and there was a trivial effect (η^2^_p_ = 0.007). There was a significant main effect for muscle (*p* = 0.008; Table 3) with a large effect (η^2^_p_ = 0.660). There was a significant difference between the left rectus femoris muscle and the left bicep femoris muscle (*p* = 0.022; Table 3). There was a significant difference between the right rectus femoris muscle and the right bicep femoris muscle (*p* = 0.018; Table 3).

### Multivariate Correlative Analysis

Sprint 1: There was no significant linear relationship between muscle desaturation rate or change in oxygen and max speed (R^2^ = 0.676), but there was a significant non-linear correlation between max speed and rectus femoris desaturation rate (tau = 0.643; *p* = 0.031), change in SmO_2_ (tau = 0.786; *p* = 0.006), bicep femoris desaturation rate (tau = 0.643; *p* = 0.031) and change in SmO_2_ (tau = 0.714; *p* = 0.014).

There was no significant non-linear relationship between muscle desaturation rate or change in oxygen and max acceleration (*p* > 0.05), but there was a significant linear correlation (R^2^ = 0.940; *p* = 0.035) between max acceleration and rectus femoris desaturation rate (coefficient = 2.204; *p* = 0.018), bicep femoris desaturation rate (coefficient = 0.262; *p* = 0.043) and change in SmO_2_ (coefficient = 0.198; *p* = 0.021).

There was no significant linear relationship between muscle desaturation rate or change in oxygen and time to max acceleration (R^2^ = 0.892), but there was a significant non-linear correlation between time to max acceleration and rectus femoris change in SmO_2_ (tau = 0.567; *p* = 0.050) and bicep femoris change in SmO_2_ (tau = 0.794; *p* = 0.007).

There were no other significant linear or non-linear relationships between muscle desaturation rate or change in oxygen and sprint metrics in sprint 1.

Sprint 2: There were no significant linear or non-linear relationships between muscle desaturation rate or change in oxygen and sprint metrics in sprint 2.

## 4. Discussion

Football is a sport that is intermittent in nature, and understanding the demands of repeated sprint activities on players is critical to improving performance [19]. In the current study we demonstrate that there is a decline in acceleration performance measured by GPS even though max speed is maintained across both 30 m sprints. The observed decline in acceleration performance without a decrease in maximum speed suggests a specific impairment in force production during the initial phase of sprinting. This pattern likely reflects an altered calcium handling and cross-bridge cycling efficiency [20] rather than a complete metabolic failure or neural inhibition. The longer time delay to fast desaturation in the bicep femoris during sprint 2 indicates compromised oxygen utilisation, potentially due to either reduced muscle activation or altered mitochondrial function following initial sprint activity [21].

We also demonstrate that there is a decline in the capacity of the bicep femoris muscle to use oxygen, with an increase in the time delay to fast desaturation in sprint 2 compared with sprint 1. Furthermore, the relationship between muscle oxygen and sprint performance is lost in sprint 2. After 2 × 30 m sprints, we also demonstrate the sigmoidal nature of recovery with impaired recovery dynamics in the bicep femoris muscle compared with the rectus femoris muscle. This means that, during duplicate sprint efforts with short recovery, the muscle oxygen recovery will be in a slow phase, which will have a knock-on effect on performance. Therefore, football players need better conditioning to improve muscle oxygen dynamics across multiple sprint efforts.

### 4.1. Sprint Metrics

We show that max speed was maintained during 2 × 30 m sprints with a 10 s recovery (Table 1); however, there was a significant decline in acceleration rate and time to maximum acceleration rate from sprint 1 to sprint 2 with a moderate-to-large effect (Table 1). In repeated sprint tests in football players, a slight decrement in maximum acceleration from sprint 1 to 3 has been shown and then a further decline over subsequent sprints [19]. The maximum acceleration rate during linear sprints in football players has been shown to be related to jump height, sprint performance and change-of-direction velocity [22] and failure to maintain across a match will lead to performance decrements. Following 3 × 40 m sprints with 2 min recovery between each sprint, it has been shown that the dynamics of muscle contraction are impeded with significant torque loss and degradation of twitch [23]. Given the direct relationship between muscle torque and acceleration [24], the decrement in acceleration in sprint 2 in the current study will reflect impaired torque and twitch due to insufficient recovery time. The exact mechanism is likely to reflect loss of calcium dynamics and cross-bridge compliance within the muscle [20].

### 4.2. Muscle Oxygenation

In sprint 1 there is a significant difference between left and right rectus femoris muscle time delay to fast desaturation (Table 2), with a similar trend in the bicep femoris muscle. An initial time delay for muscle oxygen desaturation has been reported previously and reflects the time taken for mitochondrial activity to increase beyond the rate of oxygen delivery [13]. Within cycle sprints there is no difference between the left or right leg for time delay to fast desaturation [13], which suggests that this is related to muscle recruitment during the running phases. Indeed, there is early activation of the rectus femoris of the contralateral leg compared with the ipsilateral leg during the initial ground contact of the run cycle [11]. In the rectus femoris muscle the time delay to fast desaturation is consistent across sprints. However, there is a significant increase in the time delay to fast desaturation in both legs for the bicep femoris muscle (Table 2). This is likely to reflect the extent of metabolic disturbance within the muscle after sprint 1 or a failure of the bicep femoris muscle to recover in the same way as the rectus femoris muscle after sprint 1. There is evidence of different mitochondrial stress responses in the vastus lateralis muscle compared with the triceps brachii muscle following sprint-based training over 2 weeks [25] and greater peripheral disturbance in the hamstring than in the quadricep [26]. However further research is needed to determine the mechanism of delay in mitochondrial activation in the bicep femoris muscle. Given that the total sprint time for sprint 2 is 5 s (Table 1), the extent of the delay in bicep femoris desaturation suggests that it is not activating the mitochondria above oxygen transport capacity in the second sprint until the sprint ends. This may be particularly important in football due to the extent of hamstring injuries [27], with further research needed on the impact of altered SmO_2_ dynamics and injury risk.

In sprint 1 there was a significant difference in the change in SmO_2_ across the sprint for the bicep femoris muscle compared with the rectus femoris muscle in both legs (Table 2). The change in SmO_2_ will reflect the extent of mitochondrial activation during the sprint as the mitochondria are net consumers of oxygen during exercise [21], with greater mitochondrial activation leading to a greater change in SmO_2_ across the sprint. This suggests a greater mitochondrial demand during sprint 1 in the hamstring muscle than in the quadricep muscle. Indeed, although not significant, there was a higher rate of deoxygenation in the bicep femoris muscle than in the rectus femoris muscle (Table 2). From EMG data it has been shown that progressive loss of bicep femoris happens after an initial sprint effort, but the rectus femoris activation does not decline until the third sprint in football players [28]; therefore, the loss of oxygen use may represent declining activation of the bicep femoris muscle due to a calcium disturbance after sprint 1 [20]. Furthermore, mitochondrial dysfunction has been shown after repeated sprints [29] in the hamstring, which may lead to lower oxygen use across repeated sprints in the hamstring muscles.

### 4.3. Relationship

During sprint 1 we demonstrate significant linear and non-linear relationships between oxygen use and max speed, max acceleration and time to max acceleration. Previously the desaturation rate has been shown to be related to max speed and total time during a sprint in professional footballers [9]. Within cycle sprints, a strong relationship between muscle oxygen use and cycle sprint performance has also been demonstrated [13]. In sprint 2 this significant relationship between oxygen use and sprint performance disappears. This is like what has been reported in cycle sprints in which a second Wingate-based sprint has a lower correlative relationship with oxygen use than the first Wingate sprint in the rectus femoris muscle [13]. Taken together, this suggests that muscle oxygen use is crucial to sprint performance and that impairment in oxygen use may limit the effectiveness of sprints during repeated high-intensity movements.

### 4.4. Recovery

There was a significantly greater polynomial rate constant for the left and right rectus femoris muscles than for the left and right bicep femoris muscles (Table 1). The linear component of the polynomial curve was significantly shorter for the left and right rectus femoris muscles than for the left and right bicep femoris muscles (Table 1), with a greater linear rate slope (Table 1). Others have fitted a monoexponential function to recovery [30], but this ignores the delay from exercise end to recovery starting and does not reflect the haemoglobin oxygen dissociation curve [14]. However, the rate constants reported for the vastus lateralis monoexponential are similar to the k values reported here (Table 3). Previous studies have linked perception of recovery to off-kinetics for heart rate and oxygen consumption [31]; this is likely related to the demand for oxygen of the skeletal muscle post-recovery. The different dynamics of recovery between the rectus femoris and bicep femoris muscles may be an issue during repeated sprints, which leads to earlier fatigue in the hamstring muscles than in the quadricep muscles. Across repeated sprints there is a declining activation of the hamstring compared with the quadricep in football players [17], which is likely due to the poorer oxygen recovery shown. This suggests a need for better hamstring conditioning within football.

### 4.5. Limitations and Practical Applications

A limitation of this study is the small sample size, although it represented 40% of the first team squad available on the day of testing. Given the variability in NIRS data, it may mean that smaller differences between the muscles are not detected. However, even when significance was not found, the magnitude of effect was large, which means important differences between the two muscles were still identified. The data reported show a difference in muscle oxygen response between the rectus femoris muscle and bicep femoris muscle during two 30 m sprints and a different recovery profile for muscle oxygen after duplicate sprints in professional football players. These findings highlight that the bicep femoris muscle is not utilising oxygen as well as the rectus femoris muscle. This offers a challenge as to how best to condition football players for the on-pitch demands of sprints [19]. This muscle disturbance occurs without an impact on max speed generated during each 30 m sprint, but there is a loss of acceleration in the second sprint compared with the first sprint. Therefore, there is a need to better understand the muscular demands of high-speed running during a match, and external load metrics may not give an accurate reflection of demand. The current study has been carried out as a single-session design, which may reflect the status of the players at that point in time. However, data collection was undertaken during an international break, which means any cumulative fatigue from continuous league matches [32] should be reduced. We have seen in professional boxers that the NIRS-derived muscle oxygen changes to exercise are similar across a 3-week period [21] which would suggest the NIRS data is an accurate reflection of muscle response to exercise in trained athletes across a period in which training is not changing. Furthermore, the current study used a 10 s recovery period, which is a 1:2.5 work-to-rest ratio, which may limit the applicability to match play. The average work-to-rest ratio from high-speed running has been shown to be 1:12, although during the most intense passages of play, this has been shown to be reduced to 1:2 [33]. Therefore, the findings provide key information on the muscular demands of intense moments in games.

### 4.6. Perspective

Football is an intermittent sprint-based sport [19], and understanding the demands of sprinting on the skeletal muscle of professional footballers is crucial to develop effective training regimes. Currently there is evidence for fatigue in players towards the end of each half [34]; however, we demonstrate that a failure to recover from the previous sprint is not manifested in the speed achieved but rather by a delay and a decrease in acceleration. This suggests that there is a disturbance in cross-bridge activation that needs to be overcome by greater fibre recruitment, which will exacerbate fatigue development [20]. Indeed, we report an altered bicep femoris oxygenation response in sprint 2 compared with that in sprint 1, which reflects the different recovery kinetics of the bicep femoris muscle compared with the rectus femoris muscle. This shows a different response in two muscles to the same exertional stimulus, which may be important considering that the bicep femoris is the most common site of hamstring injury [35] and the number of hamstring injuries occurring during linear sprinting in football [3]. Furthermore, the use of oxygen in sprint 1 is strongly associated with sprint performance, which is lost in sprint 2, which may mean a greater anaerobic demand that will impede subsequent activity. Indeed, GPS metrics decrease for up to 5 min following sprint activity in football [34]. Future research should look to explore this more in relation to game demand and improving hamstring conditioning.

## 5. Conclusions

Football players are asked to carry out high-speed running frequently during a match and during intense periods this can be increased by 125% [33]. Understanding the metabolic demands of these intense periods is crucial to preparing players for match play. Future studies should look to utilise NIRS on training days to explore the impact of less controlled movements on muscular demand. Hamstring injury is the biggest reason for loss of play in football, and future studies should seek to explore the role of muscle oxygenation in hamstring injury. This data suggests that conditioning of the hamstring muscles may be insufficient to meet the demands of games. There is a need to determine the impact of different training modalities on hamstring oxygenation. In conclusion, football players need to be able to sprint, and understanding the muscle response will help improve conditioning and potentially reduce injury risk.

## Figures and Tables

**Table 1:** Performance metrics during duplicate 30 m sprints.

	Sprint 1	95% CI Limits	Sprint 2	95% CI Limits
Time (s)	4.8 ± 0.7	4.3:5.3	5.0 ± 1.1	4.2:5.8
Max speed (m.s^−1^)	8.4 ±0.3	8.3:8.6	8.4 ± 0.4	8.2:8.7
Max acceleration (m.s^−2^)	5.0 ± 1.5	4.0:6.0	3.7 ± 1.2 *^b^	2.9:4.5
Time to max acceleration (s)	1.0 ± 0.3	0.8:1.2	1.8 ± 0.8 *^a^	1.2:2.4
Ave acceleration (m.s^−2^)	1.4 ± 0.2	1.3:1.5	1.3 ± 0.3 *^b^	1.1:1.5
Metabolic load (W.kg^−1^)	89.4 ± 9.0	83.2:95.7	84.8 ± 12.9 ^b^	75.9:93.8
Max deceleration (m.s^−2^)	−6.4 ± 3.4	−8.8:−4.1	−5.6 ± 1.2 ^c^	−6.4:−4.7
Ave deceleration (m.s^−2^)	−1.9 ± 0.3	−2.1:−1.7	−2.5 ± 0.3 ^b^	−2.7:−2.3

* *p* < 0.5 sprint 1 compared with sprint 2. ^a^ = large effect between sprint 1 and 2; ^b^ = moderate effect between sprint 1 and 2; ^c^ = small effect between sprint 1 and 2.

**Table 2:** Skeletal muscle oxygenation response during duplicate sprints.

	Sprint 1	95% CI Limits	Sprint 2	95% CI Limits
Right Rectus Femoris				
Time delay (s)	1.63 ± 1.28	0.6:2.9	2.72 ± 2.29	0.8:5.0
Fast desaturation (%.s^−1^)	−4.67 ± 2.68	−6.9:−2.4	−4.44 ± 2.75	−6.7:−2.1
Change in SmO_2_ (%)	23.36 ± 14.77	11.0:35.7	20.49 ± 11.11	11.2:29.8
Left Rectus Femoris				
Time delay (s)	3.87 ± 3.11 ^ǂ^	1.3:7.1	2.57 ± 1.92	1.0:4.5
Fast desaturation (%.s^−1^)	−4.34 ± 3.37	−7.2:−1.0	−5.22 ± 4.04	−8.6:−1.2
Change in SmO_2_ (%)	21.96 ± 15.88	8.7:37.8	25.39 ± 22.26	6.8:47.7
Right Bicep Femoris				
Time delay (s)	1.57 ± 1.17	0.6:2.7	5.20 ± 2.25 *	3.3:7.5
Fast desaturation (%.s^−1^)	−6.52 ± 2.38	−8.5:−4.5	−4.48 ± 3.40	−7.3:−1.6
Change in SmO_2_ (%)	37.00 ± 14.74 **	24.7:49.3	23.54 ± 17.66	8.8:38.3
Left Bicep Femoris				
Time delay (s)	2.56 ± 1.94	0.9:4.5	5.20 ± 3.06 *	2.6:8.3
Fast desaturation (%.s^−1^)	−7.37 ± 2.90	−9.8:−4.5	−4.46 ± 3.24	−7.2:−1.2
Change in SmO_2_ (%)	39.44 ± 15.48 **	26.5:54.9	25.93 ± 18.21	10.7:44.1

* *p* < 0.05 sprint 1 compared with sprint 2. ** *p* < 0.05 rectus femoris muscle compared with bicep femoris muscle during the same sprint and in the same leg. ^ǂ^ *p* < 0.05 right rectus femoris muscle compared with left rectus femoris muscle.

**Table 3:** Skeletal muscle oxygenation response during recovery from duplicate sprints.

	Right Rectus Femoris	Left Rectus Femoris	Right Bicep Femoris	Left Bicep Femoris
Polynomial				
k (%.s^−1^)	0.13 ± 0.05 **	0.14 ± 0.06 **	0.11 ± 0.05	0.11 ± 0.06
95% CI limits	0.08:0.17	0.09:0.19	0.07:0.15	0.06:0.16
L (%)	43.85 ± 17.85	43.81 ± 13.70	41.92 ± 13.50	52.07 ± 17.88
95% CI limits	27.8:55.8	32.4:55.3	30.6:53.2	37.1:67.0
Time 50% (s)	25.23 ± 11.52	22.34 ± 6.35	29.25 ± 9.69	21.68 ± 5.21
95% CI limits	16.3:35.3	17.0:27.7	21.1:37.4	17.3:26.0
Linear				
Linear fast recovery (%.s^−1^)	1.13 ± 0.34	1.22 ± 0.46	0.94 ± 0.20	1.08 ± 0.29
95% CI limits	0.86:1.43	0.83:1.61	0.78:1.11	0.84:1.33
Time linear fast recovery (s)	26.99 ± 10.61 **	28.48 ± 10.48 **	36.13 ± 14.81	35.73 ± 13.44
95% CI limits	18.1:35.9	19.7:37.2	23.8:48.5	24.5:46.9

k is the rate constant describing the steepness of the curve; L is the change in SmO_2_ across the curve. ** *p* < 0.05 rectus femoris muscle compared with bicep femoris muscle during the same sprint and in the same leg.

## Data Availability

Data is available upon reasonable request by contacting the corresponding author.

## Abbreviations

The following abbreviations are used in this manuscript:

NIRS	near-infrared spectroscopy
VO_2_ max	maximal oxygen consumption
SmO_2_	muscle oxygen saturation
GPS	global positioning system
